# Closing Critical Gaps in the 2026 Bundibugyo Ebola Response: Five Actionable Priorities

**DOI:** 10.7759/cureus.111921

**Published:** 2026-07-01

**Authors:** Prasanta R Mohapatra, Baijayantimala Mishra

**Affiliations:** 1 Pulmonary Medicine and Critical Care, All India Institute of Medical Sciences, Bhubaneswar, Bhubaneswar, IND; 2 Microbiology, All India Institute of Medical Sciences, Bhubaneswar, Bhubaneswar, IND

**Keywords:** bundibugyo, congo, ebola, uganda, virus

## Abstract

The 2026 Bundibugyo virus disease (BDBV) outbreak in the Democratic Republic of the Congo and adjacent Uganda has revealed major deficiencies in outbreak response. The available diagnostics, vaccines, and therapies were primarily designed against Zaire Ebola virus - leaving us ill-prepared for BDBV detection and control. We call for five immediate actions to strengthen existing efforts. First, rapidly establish a decentralized three-tier molecular diagnostic network to allow timely detection and genomic surveillance. Second, since there are few approved treatments that target BDBV specifically, adaptive platform trials should assess the potential of repurposed host-directed therapies. Third, in conflict-affected settings, routine enabling environment data collection to strengthen surveillance is needed, whilst digital proximity tracing and social support mechanisms, such as community tracing collaborative (CTC) responses to combat community transmission through contact identification and isolation, would also add value. Fourth, ring vaccination strategies with recombinant vesicular stomatitis virus-Zaire Ebola virus (rVSV-ZEBOV) as a post-exposure intervention should be explored, given the potential for cross-protection against BDBV. Fifth, implementation of extensive genomic surveillance should be enhanced to differentiate between continuous human transmission and sporadic zoonotic spillover events while informing ecology-based control strategies. Together, these recommendations highlight actionable, scalable steps that can be implemented quickly through existing systems and out-of-the-box solutions. Filling these key gaps could significantly affect the outcome of this current outbreak and enhance regional and global preparedness for future ebolavirus outbreaks.

## Editorial

The recent rapid, uncontrolled spread of the Bundibugyo virus in the Democratic Republic of the Congo (DRC) and neighboring Uganda (as of 24 June 2026, over 1024 confirmed cases and 277 deaths [1]) has created a Public Health Emergency of International Concern (PHEIC), highlighting significant challenges to global health preparedness alongside deep humanitarian challenges [1]. The WHO declared the Bundibugyo virus disease (BDBV) outbreak a Public Health Emergency of International Concern on 17 May 2026 [2]. It has become the third recognized emergence of this orthoebolavirus species. During prior outbreaks of 2007 and 2012, the world was unprepared [3]. We describe five feasible interventions that should substantially alter the trajectory of this and future outbreaks.

First, the initial delay and partial failure in identifying the virus are likely due to an unfortunate system in place. Because the widely deployed GeneXpert (cartridge-based closed reverse transcription polymerase chain reaction (RT-PCR)) platform (Cepheid, California, USA) was designed to detect Zaire Ebola virus, initial samples from the outbreak tested negative, delaying detection by weeks. Because all diagnostics (e.g., GeneXpert), vaccines (e.g., recombinant vesicular stomatitis virus-Zaire Ebola virus (rVSV-ZEBOV)), and therapeutics were developed specifically for Zaire Ebola virus rather than Bundibugyo virus, transmission chains were likely undetected, a scenario that exposes the fundamental flaw of designing diagnostics solely for the pathogens. Recent confirmation issues required the sample to be transported nearly 2,900 km from Ituri to Kinshasa, resulting in diagnostic delays of 7-14 days despite logistical challenges [4]. Hence, it is prudent and desirable to deploy a tiered, decentralized, adjacent-to-patient-locality molecular diagnostic network as soon as feasible and support loco-regional laboratory networks.

A Tier-1 laboratory can be near outbreak epicenters; it is needed to deploy RADIONE pan-ebolavirus RT-PCR kits (KH Medical Co. Ltd., South Korea) to provincial hospitals. These are needed to detect BDBV but require validation for sensitivity/specificity [4]. But it is essential to establish a three-tier system. Tier-2 laboratories can be located in border zones. They may include mobile polymerase chain reaction (PCR) laboratories using portable Oxford Nanopore Technologies (ONT) (Oxford Science Park, Oxford, UK) or MinION platforms (Oxford Science Park, Oxford, UK) at major crossing points among the DRC, Uganda, Rwanda, and Burundi. A Tier-3 national referral laboratory should be reserved for centralized sequencing for quality assurance and phylogenetic monitoring only, not for primary diagnosis. It can be done by repurposing existing Ebola diagnostic networks from the 2018-2020 North Kivu outbreak, where mobile laboratories were successfully deployed despite active conflict [5]. 

Second, as we know, there is no approved BDBV-specific antiviral. Monoclonal antibodies effective against Zaire Ebola virus (e.g., Ebanga, REGN-EB3) do not bind BDBV due to ~30% genomic divergence in the glycoprotein [3]. It will certainly take time to make countermeasures, which may be months to years away. Hence, it is desired to prepare the platform for adapting clinical trials for antivirals, vaccines, monoclonal antibodies, and repurposed host-directed optimal therapies at the earliest.

It is suggested that an adaptive, multi-arm platform trial be initiated to evaluate repurposed host-directed therapies that modulate the syndemic interaction observed in the field. Recent fieldwork in Bunia identified concurrent bacterial sepsis (positive blood cultures within eight hours) and high malaria parasitaemia in fatal cases, suggesting a lethal syndemic interaction rather than intrinsic viral virulence alone [6]. Repurposing drugs like atorvastatin (pleiotropic anti-inflammatory and endothelial-stabilizing effects) and low-dose corticosteroids (dexamethasone 6 mg daily) may attenuate cytokine storm without full immunosuppression; these may be used for the time being as needed. Antibiotic coverage may be added for bacterial co-infection as per the culture result.

Third, contact follow-up in Ituri province is only 21%, and armed conflict prevents traditional house-to-house tracing [7]. Displacement causes exposed individuals to move before diagnosis and can create silent transmission chains. It is essential to implement community-based surveillance with digital proximity tracing in conflict zones by deploying a low-tech, privacy-preserving proximity tracing system using existing feature phones via Bluetooth metadata logging (no Global Positioning System (GPS), no personal identifiers). This approach was successfully used for Ebola in DRC in 2019, and it can be reactivated within days [4].

For this, community health workers distribute coded tokens or install lightweight apps voluntarily. When a case is confirmed, historical proximity logs from the previous 21 days are extracted from the phones of consenting contacts. Tracing alerts are delivered via existing village health team networks, not by security forces (which are critical for trust). This must be paired with cash transfers for voluntary isolation compliance ($20-50/week), which reduced resistance in the 2018-2020 outbreak [8].

Fourth, as there is no licensed BDBV vaccine, animal models show that rVSV-ZEBOV (Ervebo) provides heterologous protection against BDBV despite ~30% glycoprotein divergence, likely through cross-reactive T-cell epitopes [1]. A ring vaccination trial using rVSV-ZEBOV in a post-exposure prophylaxis design, similar to the 2015 Guinea trial that established Ervebo's efficacy, should be launched [9]. Rings can be defined as contacts and contacts-of-contacts of newly confirmed BDBV cases (expected number 50-150 per ring). Immediate vaccination of all consenting ring members should be implemented (no placebo - ethical given animal efficacy data). Attack rates can be compared to historical rings from the 2007 and 2012 outbreaks (nested case-control design). The WHO has already convened emergency consultations on vaccine candidates. The rVSV platform has existing manufacturing capacity and safety data in pregnant women and children. Doses can be redirected from Zaire-focused stockpiles.

Finally, genomic surveillance for transmission chain mapping is critical. Preliminary analysis of three near-complete BDBV genomes suggests that there are multiple independent zoonotic spillover events, not a single introduction [10]. This fundamentally changes the response strategy: if true, resources must be allocated to preventing repeated spillovers, not just interrupting human transmission. Therefore, the doable solution is to sequence all confirmed cases as early as possible using amplicon-based nanopore protocols (which cost ~$20-30/sample) [11]. The data can be shared immediately via an open-source database that makes it convenient for researchers and public health workers to share, review, and act on viral pathogen genomic data - for example, Pathoplexus (not GenBank, which has slower release mechanisms). It is required to generate real-time phylogenetic trees to distinguish between a single introduction with onward transmission (target contact tracing) and multiple independent spillovers (target ecological source investigation).

If multiple spillovers are confirmed, deploy bat ecology teams to mine sites (such as the Mongwalu gold mining area) and to human dwellings where *Mops condylurus* (Angolan free-tailed bat), the known BDBV reservoir, roosts. It is essential to identify and close specific human-bat contact points (e.g., removing bats from ceilings, covering water sources) [12].

Hence, rapid diagnostics, supportive life-saving care, coordinated regional genomic surveillance, infection control, and faster action at every step within trusted community partnerships, along with protection of frontline healthcare workers and uninterrupted logistics, remain essential.

## References

[1] Cassasus B (2026). Ebola: France announces first case as doctor tests positive after returning from DRC. BMJ.

[2] Haider N, Khan RA, Rahman KM (2026). Beyond past Ebola outbreaks: delayed detection, preparedness gaps, and the vaccine race during the 2026 Bundibugyo virus outbreak. Int J Infect Dis.

[3] Sullivan NJ (2026). Bundibugyo virus disease in 2026 - clinical and public health responses. N Engl J Med.

[4] Karim SA, Mahomed S, Lewis L, Karim SS (2026). Urgent need for a reliable rapid diagnostic test for the Ebola epidemic caused by Bundibugyo virus in Africa. Lancet.

[5] Mambu F, Bulabula J, Edidi F (2023). Contribution of the mobile laboratory to the fight against infectious diseases: the case of Ebola virus disease. (Article in French). Med Mal Infect Form.

[6] Tonen-Wolyec S, Bélec L (2026). The 17th Ebola outbreak in the Democratic Republic of the Congo: a syndemic challenge. Lancet.

[7] Chamla D, Belizaire MR, Co IF, Jinadu A, Mamadu I, Atagbaza AO (2026). Size of the 2026 Ebola outbreak and risk of cross-border spillover from Bundibugyo virus in Ituri Province, DR Congo, and its implications for preparedness: a recalibrated stochastic modelling study. Lancet Infect Dis.

[8] Keita M, Polonsky JA, Ahuka-Mundeke S (2023). A community-based contact isolation strategy to reduce the spread of Ebola virus disease: an analysis of the 2018-2020 outbreak in the Democratic Republic of the Congo. BMJ Glob Health.

[9] Henao-Restrepo AM, Longini IM, Egger M (2015). Efficacy and effectiveness of an rVSV-vectored vaccine expressing Ebola surface glycoprotein: interim results from the Guinea ring vaccination cluster-randomised trial. Lancet.

[10] Hulseberg CE, Kumar R, Di Paola N (2021). Molecular analysis of the 2012 Bundibugyo virus disease outbreak. Cell Rep Med.

[11] Mwenda M, Mosler K, Bohmeier B (2026). Continental-scale genomic surveillance of Plasmodium falciparum malaria across sub-Saharan Africa with rapid nanopore sequencing. Nat Commun.

[12] Goldstein T, Anthony SJ, Gbakima A (2018). The discovery of Bombali virus adds further support for bats as hosts of ebolaviruses. Nat Microbiol.

